# Association between preventive health management and high-risk drinking among women of childbearing age: a correlational study of the ninth Korea National Health and Nutrition Examination Survey

**DOI:** 10.4069/whn.2025.12.08

**Published:** 2025-12-31

**Authors:** Hee Jeong Lee, Hye Young Kim

**Affiliations:** College of Nursing, Keimyung University, Daegu, Korea

**Keywords:** Alcohol drinking, Binge drinking, Health behavior, Preventive health services, Women

## Abstract

**Purpose:**

This study aimed to investigate the prevalence of high-risk drinking among women of childbearing age and to identify factors associated with high-risk drinking.

**Methods:**

Data were obtained from the first (2022) and second (2023) years of the ninth Korea National Health and Nutrition Examination Survey. The study sample comprised 2,101 women of childbearing age (19–49 years). Data were analyzed using complex sample analysis to appropriately account for population weights, survey clustering, and stratified sampling.

**Results:**

Among respondents, 9.5% were classified as high-risk drinkers. Significant differences in alcohol consumption were observed according to education level, employment status, skipping breakfast in the past 2 days, physical activity intensity, perceived stress level, smoking status, and dental check-up status. In the multiple logistic regression analysis, education level (odds ratio [OR]=1.26, *p*=.015), employment status (OR=1.44, *p*=.045), skipping breakfast in the past 2 days (OR=1.81, *p*<.001), smoking status (OR=4.20, *p*<.001), and dental check-up status (OR=1.70, *p*=.002) remained significantly associated with high-risk drinking.

**Conclusion:**

These findings highlight the need for targeted public health strategies to reduce high-risk drinking among women of childbearing age. Healthcare providers may use these findings to design interventions that strengthen preventive health behaviors, promote regular meal patterns, support smoking cessation, and encourage routine dental check-ups. Public health practitioners may also utilize these results to develop programs that promote healthy lifestyle practices in this population. Further research is needed to refine and enhance intervention strategies.

## Introduction

Globally, increasing alcohol consumption has exacerbated health problems and social burdens [1]. In particular, rising alcohol consumption among women has emerged as a significant public health concern [2,3]. According to the 2022 National Health Statistics, the prevalence of high-risk drinking among women—defined as consuming five or more drinks per occasion on 2 or more days per week—was highest among women in their 20s (8.8%) and 30s (9.3%), underscoring high-risk alcohol use among women of childbearing age as a critical issue [4]. In contrast, when drinking patterns are compared by sex and age, men in their 40s demonstrate the highest rates of high-risk drinking, reflecting clear differences in drinking patterns across sex and age groups [5].

Women of childbearing age are in a critical life stage during which pregnancy may be possible or planned. Health management during this period can substantially influence fetal development and overall family well-being [6,7]. At the individual level, alcohol use can adversely affect women’s reproductive health by reducing fertility and increasing the risk of menstrual irregularities [8]. Alcohol consumption is also associated with high-risk sexual behaviors, which may indirectly increase the risk of sexually transmitted infections, including human immunodeficiency virus and human papillomavirus [9]. In the context of pregnancy, alcohol use is linked to adverse maternal and fetal outcomes, and even moderate intake has been shown to increase the risk of fetal growth restriction and low birth weight, raising concerns about long-term fetal development [7]. Furthermore, alcohol consumption among women of childbearing age may affect family functioning, as drinking-related behaviors can contribute to emotional strain, relationship conflict, and reduced overall family well-being [10]. Taken together, these interconnected effects indicate that alcohol consumption in this population represents an important public health concern.

Given these concerns, preventive health management among women of childbearing age warrants particular attention [11]. Preventive health behaviors are fundamental to disease prevention and health promotion and play an important role in preparing for healthy pregnancy and childbirth in this population [12]. Regular health check-ups and vaccinations contribute to the maintenance of overall health and the protection of maternal and fetal health before and after pregnancy [13,14]. In addition, a healthy diet and regular physical activity can help mitigate health risks associated with alcohol consumption and support the maintenance of a healthy lifestyle [15].

Previous studies on women’s alcohol consumption have shown that drinking behavior is influenced by multiple factors [2]. Stress, social environment, mental health, and cultural factors have been consistently identified as major determinants [3,16]. Stress, in particular, has been recognized as a key driver of alcohol consumption among women, often arising from pressures related to work and family responsibilities [17]. Social environments, including prevailing drinking cultures, further shape women’s drinking patterns, while mental health conditions such as depression and anxiety are strongly associated with high-risk drinking [3,18]. In addition, increasing economic participation and growing awareness of gender equality in modern society have been closely linked to changes in women’s drinking patterns [3]. However, most prior studies have focused primarily on the causes of alcohol use or drinking patterns themselves, whereas research explicitly examining the relationship between preventive health management and high-risk drinking remains limited [2,18]. Preventive health behaviors may function not only as isolated health-promoting actions but also as moderating factors that influence the likelihood or severity of risky behaviors, including high-risk drinking [9]. Individuals who regularly engage in health-promoting behaviors tend to be more aware of their overall health status, which may reduce tendencies toward harmful alcohol use [19]. Conversely, limited engagement in preventive care may reflect a broader pattern of risk-prone behaviors, including problematic drinking [2]. Supporting this perspective, a recent study reported that reduced oral hygiene practices, such as infrequent tooth brushing and limited dental check-ups, were significantly associated with increased alcohol consumption and unhealthy dietary habits among university students [20]. Regular general health check-ups, dental check-ups, vaccinations, and healthy dietary behaviors are therefore essential components of preventive health management that may help mitigate alcohol-related health risks. Examining these factors is critical for understanding the interaction between drinking behavior and health management among women of childbearing age [9,19].

This study aimed to examine the association between high-risk drinking behavior and preventive health management among women of childbearing age using data from the Korea National Health and Nutrition Examination Survey (KNHANES). By addressing this relationship, the study seeks to contribute to efforts to promote health among women of childbearing age and to prevent adverse outcomes related to alcohol consumption. Ultimately, the findings are intended to provide a foundation for the development of tailored intervention strategies aimed at reducing alcohol use and safeguarding the well-being of women of childbearing age, their fetuses, and their families.

## Methods

**Ethics statement:** This study did not involve sensitive information. Because the survey was conducted anonymously, the requirement for obtaining prior consent from the participants was waived by the Institutional Review Board of Keimyung University (40525-202502-HR-101-01).

### Study design

This correlational study used data from the ninth KNHANES to examine the association between preventive health behaviors and high-risk drinking among women of childbearing age. The study was reported in accordance with the STROBE guidelines (https://www.strobe-statement.org/).

### Sample and sampling

**Figure 1.**
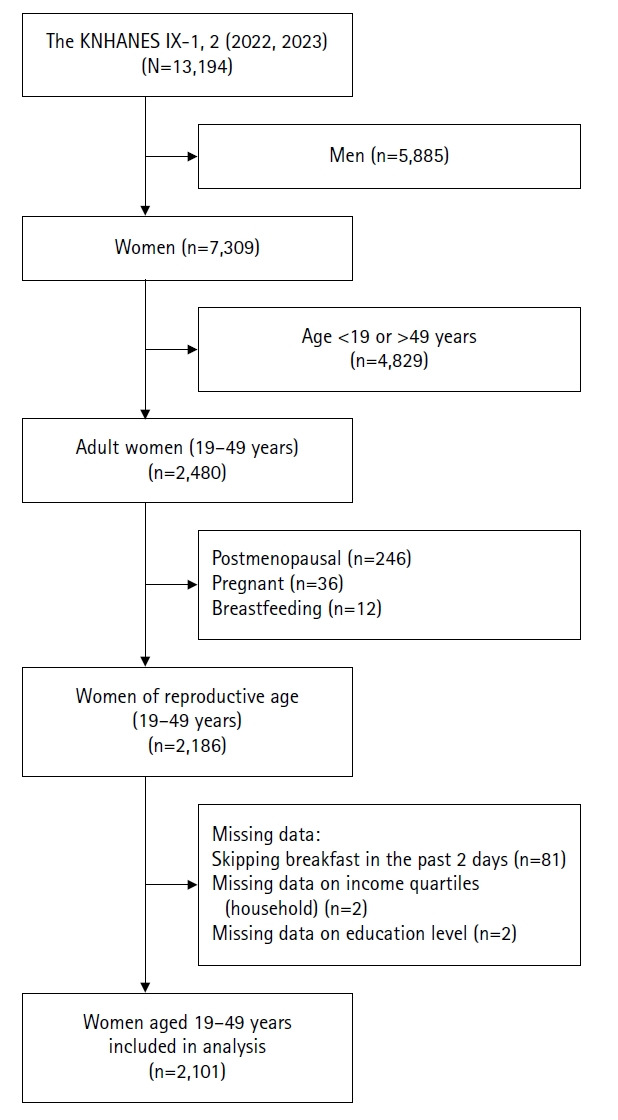
Flowchart of the study population. Preventive health behaviors and alcohol consumption among women of childbearing age using data from the first and second years (2022 and 2023) of the ninth Korea National Health and Nutrition Examination Survey (KNHANES), conducted by the Korea Disease Control and Prevention Agency.

This study utilized data from the first and second years (2022 and 2023) of the ninth KNHANES, conducted by the Korea Disease Control and Prevention Agency. The study population consisted of women of childbearing age between 19 and 49 years. Postmenopausal women, pregnant or breastfeeding women, and participants with missing values for key variables were excluded. As a result, the final analytic sample comprised 2,101 participants (Figure 1). Variables used in this study were selected from the KNHANES health examination and nutrition survey components.

### Variables

#### General characteristics

Participants’ age, average monthly household income, education level, employment status, marital status, and pregnancy experience were examined. Age was categorized into three groups: 19–29, 30–39, and 40–49 years. Household income was classified into four quartiles based on average monthly household income: low, lower-middle, upper-middle, and high. Educational level was categorized as high school graduate or less and college graduate or higher. Employment status was classified as employed or unemployed. Marital status was categorized as married or unmarried, and pregnancy experience was classified as having experienced pregnancy or not.

#### Preventive health behaviors

To assess preventive health behaviors, participants’ lifestyle habits, mental health status, smoking behavior, and health check-up participation were examined. Lifestyle habits included skipping breakfast during the past 2 days, as recorded in the dietary intake survey, and physical activity intensity. Physical activity intensity was classified according to the 2020 World Health Organization physical activity guidelines [21]. The number of days and duration of moderate-intensity physical activity per week were calculated, and participants engaging in at least 600 metabolic equivalent of task (MET)-minutes per week were classified as having high physical activity, whereas those below this threshold were categorized as having low physical activity [21]. Perceived stress level was assessed using self-reported responses regarding the level of stress experienced in daily life and was categorized into two groups: low stress and high stress. Smoking status was determined based on current smoking behavior, including both conventional and electronic cigarettes, and participants were classified as smokers or nonsmokers.

Finally, indicators of health check-up participation included influenza vaccination within the past year, cancer screening within the past 2 years, general health check-up status (defined as having undergone a routine health examination within the past 2 years), and dental check-up participation within the past year.

#### Alcohol consumption

Alcohol consumption was assessed based on drinking frequency during the past year. Women who did not consume alcohol during the past year were classified as nondrinkers. Moderate drinkers were defined as individuals who had consumed alcohol at least once during the past year but did not meet the criteria for high-risk drinking. High-risk drinkers were classified according to the definition of high-risk drinking, which included individuals who reported consuming five or more drinks per occasion at least twice per week [1].

### Data collection and analysis

Data were analyzed using IBM SPSS Statistics for Windows, ver. 21 (IBM Corp., Armonk, NY, USA). Complex sample analysis was performed to account for population weights, survey clustering, and stratified sampling. The distributions of participants’ general characteristics and key variables were summarized using frequencies and percentages. Differences between key variables were examined using cross-tabulation and the chi-square test.

To evaluate the association between preventive health behaviors and high-risk drinking behavior, multiple logistic regression analysis was conducted, and results were presented as odds ratios (ORs) with 95% confidence intervals (CIs). All variables included in the descriptive analyses were entered simultaneously into the regression model as independent variables.

## Results

### Alcohol consumption in women of childbearing age

Among women of childbearing age, 17.5% were classified as nondrinkers, 73.0% as moderate drinkers, and 9.5% as high-risk drinkers (Table 1).

### Differences in alcohol consumption based on general characteristics and preventive health behaviors in women of childbearing age

To compare alcohol consumption according to general characteristics and preventive health behaviors among women of childbearing age, nondrinkers and moderate drinkers were combined into a non–high-risk drinking group and compared with high-risk drinkers. The analysis revealed significant differences between the two groups with respect to education level, employment status, skipping breakfast in the past 2 days, physical activity intensity, perceived stress level, smoking status, and dental check-up status.

Among education levels, women with a high school graduate education or less had a higher proportion of high-risk drinkers (14.6%) than those with a college graduate education or higher (7.9%), and this difference was statistically significant (*χ*^2^=11.04, *p*<.001). Employment status also differed significantly, with employed women showing a higher proportion of high-risk drinkers, whereas unemployed women exhibited a relatively lower proportion (*χ*^2^=6.50, *p*=.013) (Table 2).

High-risk drinking was more prevalent among women who reported skipping breakfast in the past 2 days (*χ*^2^=29.29, *p*<.001). Physical activity intensity was also significantly associated with high-risk drinking. Women with high physical activity intensity (≥600 MET-min/week) showed a higher proportion of high-risk drinking (14.3%) compared with those with low physical activity intensity (<600 MET-min/week; 9.6%) (*χ*^2^=5.01, *p*=.004). Additionally, women who perceived higher stress levels demonstrated a lower proportion of high-risk drinking compared with those reporting lower stress levels (*χ*^2^=10.61, *p*=.004), whereas smokers exhibited a markedly higher proportion of high-risk drinking than nonsmokers (*χ*^2^=118.48, *p*<.001). No significant differences were observed between high-risk and non–high-risk drinkers with respect to general health check-up status, cancer screening status, or influenza vaccination status. However, a significant difference was identified for dental check-up status, with a higher proportion of high-risk drinkers among women who had not undergone a dental check-up (*χ*^2^=14.11, *p*<.001) (Table 3).

### Factors associated with high-risk drinking based on preventive health behaviors in women of childbearing age

In the multiple logistic regression analysis, high-risk drinking behavior was significantly associated with education level, employment status, skipping breakfast in the past 2 days, smoking status, and dental check-up status. Women with a high school graduate education or less were 1.26 times more likely to belong to the high-risk drinking group than those with a college graduate education or higher (*p*=.015). Employed women were 1.44 times more likely to be classified as high-risk drinkers than unemployed women (*p*=.045). Women who skipped breakfast in the past 2 days had a 1.81-fold higher likelihood of high-risk drinking compared with those who did not skip breakfast (*p*<.001). Smokers were 4.20 times more likely to engage in high-risk drinking than nonsmokers (*p*<.001). In addition, women who did not undergo dental check-ups were 1.70 times more likely to be classified as high-risk drinkers compared with those who did (*p*=.002). In contrast, perceived stress level and physical activity intensity were not significantly associated with high-risk drinking behavior (Table 4).

## Discussion

This study analyzed the relationship between preventive health behaviors and high-risk drinking among women of childbearing age. Factors associated with high-risk drinking were categorized into two domains: general characteristics, including education level and employment status, and preventive health behaviors, such as smoking status, skipping breakfast, and dental check-up participation. Among all participants, the overall alcohol consumption rate was 82.5%, with 73.0% identified as moderate drinkers and 9.5% as high-risk drinkers. In the multiple logistic regression analysis, smoking status, skipping breakfast in the past 2 days, dental check-up status, employment status, and education level were significantly associated with high-risk drinking behavior.

Smoking status showed the strongest association with high-risk drinking, with current smokers being approximately four times more likely to engage in high-risk drinking than nonsmokers. This finding is consistent with previous research indicating that smoking and drinking frequently co-occur as clustered health-risk behaviors [5,18]. Furthermore, this co-occurrence has been reported to be particularly pronounced among women in their 20s and 30s [22]. Taken together, these findings support the need for integrated intervention programs that simultaneously address both high-risk drinking and smoking behaviors.

Skipping breakfast in the past 2 days also demonstrated a notable association with high-risk drinking. Irregular eating patterns may reflect broader profiles of unhealthy behaviors, consistent with prior evidence showing that irregular dietary habits are linked to risky behaviors, including problematic alcohol use [2,23]. Accordingly, interventions aimed at improving lifestyle behaviors should emphasize the importance of regular meals, particularly breakfast, as part of an integrated approach to health management.

Dental check-up status was another significant predictor, with women who did not undergo dental check-ups being more likely to engage in high-risk drinking. This finding suggests that dental check-up participation may reflect not only attention to oral health but also broader attitudes toward preventive health management [20]. Previous studies have shown that individuals who do not participate in preventive health services are less likely to engage in healthy behaviors and often have limited awareness of the need for ongoing health management [24,25]. In addition, prior research has demonstrated a significant association between high-risk drinking and periodontal disease, with alcohol consumption potentially exacerbating oral health problems by impairing immune responses and accelerating periodontal tissue damage [26]. For women of childbearing age, dental check-ups may therefore provide valuable opportunities for nurses to identify risky drinking patterns early and to guide patients toward appropriate counseling or support services.

Employment status was also associated with high-risk drinking, with employed women demonstrating a higher likelihood of hazardous alcohol use. This finding aligns with previous research indicating that gender norms and workplace environmental factors play an important role in shaping drinking patterns among female employees [3]. Moreover, qualitative studies have reported that young workers often use alcohol as a means of coping with work-related stress and as a tool for maintaining social relationships within workplace culture [16]. These findings underscore the importance of workplace-centered interventions that address stress management and organizational drinking norms.

Education level demonstrated the weakest, yet still statistically significant, association with high-risk drinking. Women with lower educational attainment were more likely to engage in high-risk drinking, which is consistent with earlier findings that health-risk behaviors such as depression, smoking, and binge drinking often cluster together [2]. These results highlight the importance of tailoring alcohol-related interventions according to educational background and health literacy levels.

In contrast, influenza vaccination status, participation in general health check-ups, participation in cancer screening programs, perceived stress level, and physical activity intensity were not significantly associated with high-risk drinking in the multivariate analysis. Although some of these variables showed significant differences in univariate analyses, their associations may have been attenuated by stronger predictors such as smoking status. Participation in national screening programs may not fully capture individual health attitudes or motivations, which could weaken their observed relationship with drinking patterns. Previous research has also suggested that physical activity may occur in social contexts where alcohol consumption is common or may function as a coping strategy for stress, indicating a more complex relationship with alcohol use [27]. These findings underscore the importance of including theoretically relevant variables in analyses, even when they do not retain statistical significance in multivariate models.

This study has several limitations. Because cross-sectional KNHANES data were used, causal relationships between preventive health behaviors and high-risk drinking could not be established. In addition, reliance on self-reported data introduces the possibility of underreporting or overreporting alcohol consumption and preventive health behaviors due to recall bias or social desirability bias. Future studies should employ longitudinal designs to clarify causal pathways linking preventive health behaviors with high-risk drinking and should consider linking survey data with objective health check-up records to improve measurement accuracy. Qualitative research exploring the mechanisms underlying non-significant associations may also provide valuable insights into the complex patterns of high-risk drinking and health behaviors.

Despite these limitations, this study contributes to a more nuanced understanding of the relationship between high-risk drinking and preventive health behaviors among women of childbearing age. By identifying key predictors such as smoking status, skipping breakfast in the past 2 days, dental check-up status, employment status, and education level, the findings underscore the need for targeted interventions. Increasing participation in dental check-ups and using these encounters as opportunities for health counseling may be particularly effective. In addition, integrated and multifaceted intervention strategies that address complex patterns of co-occurring health behaviors are needed to prevent and manage high-risk drinking and to promote healthier lifestyles among women.

In conclusion, this study demonstrates that both preventive health behaviors and general characteristics significantly influence high-risk drinking among women of childbearing age, underscoring the need for policy-level strategies that promote healthier lifestyles and facilitate early identification of individuals at risk. The findings have several practical and policy implications. Health promotion programs targeting women of childbearing age should prioritize education about the risks of high-risk drinking, particularly among women with lower educational attainment, employed women, and smokers. Promoting dental check-up participation and incorporating brief, nurse-led alcohol use counseling into dental visits may represent a novel approach to improving both oral health and overall health management. Workplace-focused interventions addressing drinking culture and healthy eating practices may also be effective. Finally, interventions should account for the clustering of risky health behaviors, such as smoking and high-risk drinking, and implement integrated strategies to address these behaviors simultaneously.

## Figures and Tables

**Table 1.** Classification of drinking patterns in women of childbearing age (N=2,101)

Variable	n^†^ (%^‡^)	SE
Nondrinker	368 (17.5)	0.90
Moderate drinker	1,534 (73.0)	1.10
High-risk drinker	199 (9.5)	0.70

† Unweighted number.

‡ Weighted percent.

**Table 2.** Alcohol consumption according to general characteristics (N=2,101)

Variable	Categories	n^†^ (%^‡^)			χ^2^ (*p*)
		Total	Non–high-risk drinking group (n=1,902)	High-risk drinking group (n=199)	
Age (year)	≤29	594 (31.9)	534 (90.1)	60 (9.9)	4.37 (.156)
	30–39	633 (31.1)	566 (92.5)	67 (7.5)	
	≥40	874 (37.0)	802 (90.0)	72 (10.0)	
Income quartiles (household)	Lower	163 (7.8)	145 (90.0)	18 (10.0)	0.36 (.964)
	Lower–middle	475 (21.5)	433 (88.8)	42 (11.2)	
	Upper–middle	750 (35.7)	673 (90.8)	77 (9.2)	
	Upper	713 (35.0)	651 (91.3)	62 (8.7)	
Education level	High school graduate or less	419 (18.6)	353 (85.4)	66 (14.6)	11.04 (<.001)
	College graduate or higher	1,682 (81.4)	1,549 (92.1)	133 (7.9)	
Employment status	Employed	1,372 (65.8)	1,228 (89.8)	144 (10.2)	6.50 (.013)
	Unemployed	729 (34.2)	674 (93.2)	55 (6.8)	
Married	Yes	1,283 (57.7)	1162 (90.5)	121 (9.5)	.00 (.974)
	No	818 (42.3)	740 (90.5)	78 (9.5)	
Experienced pregnancy	Yes	1,201 (52.3)	1,090 (91.5)	111 (8.5)	.00 (.974)
	No	900 (47.7)	812 (90.5)	88 (9.5)	

Income quartiles: Lower, first quartile; Lower–middle, second quartile; Upper–middle, third quartile; Upper, fourth quartile.

† Unweighted number.

‡ Weighted percent.

**Table 3.** Alcohol consumption according to preventive health behaviors (N=2,101)

Variable	Categories	n^†^ (%^‡^)			χ^2^ (*p*)
		Total	Non–high-risk drinking group (n=1,902)	High-risk drinking group (n=199)	
Skipping breakfast for the past 2 days	Yes	919 (45.8)	799 (86.9)	120 (13.1)	29.29 (<.001)
	No	1,182 (54.5)	1,103 (93.3)	79 (6.7)	
Physical activity intensity	High^ ǁ^	301 (15.0)	282 (85.7)	19 (14.3)	5.01 (.004)
	Low ^ǁ^	1,800 (85.0)	1,620 (90.4)	180 (9.6)	
Perceived stress level	Low stress	1,354 (64.5)	1,242 (88.5)	112 (11.5)	10.61 (.004)
	High stress	747 (35.5)	660 (93.0)	87 (7.0)	
Smoking status	Yes	191 (9.6)	133 (70.3)	58 (29.7)	118.48 (<.001)
	No	1,910 (90.4)	1,769 (93.2)	141 (6.8)	
Vaccination status	Yes	672 (32.4)	623 (92.5)	49 (7.5)	2.91 (.110)
	No	1,429 (67.6)	1,279 (90.3)	150 (9.7)	
Cancer screening status	Yes	1,252 (57.0)	1,130 (90.6)	122 (9.4)	0.52 (.472)
	No	849 (43.0)	772 (91.5)	77 (8.5)	
General health check-up status	Yes	1,445 (67.4)	1,318 (91.8)	127 (8.2)	2.22 (.158)
	No	656 (32.6)	584 (90.4)	72 (9.6)	
Dental check-up status	Yes	1,005 (48.8)	939 (93.4)	66 (6.6)	14.11 (<.001)
	No	1,096 (51.2)	963 (88.7)	133 (11.3)	

Physical activity intensity: High, ≥600 metabolic equivalent of task (MET)-min/week; Low, <600 MET-min/week.

† Unweighted number.

‡ Weighted percent.

**Table 4.** Relationships between drinking patterns and preventive health behaviors (N=2,101)

Variable	Categories	Odds ratio (95% CI)	*p*
Education level	High school or less	1.26 (1.03–1.52)	.015
Employment status	Employee	1.44 (1.00–2.05)	.045
Skipping breakfast for the past 2 days	Yes	1.81 (1.31–2.50)	<.001
Physical activity intensity	High (≥600 MET-min/week)	1.46 (0.90–2.03)	.206
Perceived stress level	High	1.30 (0.93–1.81)	.156
Smoking status	Yes	4.20 (2.75–6.65)	<.001
Dental check-up status	No	1.70 (1.22–2.38)	.002

CI: Confidence interval; MET: metabolic equivalent of task. The reference categories were education level (college graduate or higher), employment status (unemployed), skipping breakfast in the past 2 days (no), perceived stress level (low stress), smoking status (no), physical activity Intensity (low), and dental check-up status (yes).
